# Self-Healable Supramolecular Vanadium Pentoxide Reinforced Polydimethylsiloxane-Graft-Polyurethane Composites

**DOI:** 10.3390/polym11010041

**Published:** 2018-12-29

**Authors:** Ali Sabri Berkem, Ahmet Capoglu, Turgut Nugay, Erol Sancaktar, Ilke Anac

**Affiliations:** 1 Department of Material Science and Engineering, Gebze Technical Unviersity, 41400 Kocaeli, Turkey; capoglu@gtu.edu.tr; 2 Department of Polymer Engineering, The University of Akron, Akron, OH 44325, USA; erol@uakron.edu; 3 Department of Chemistry, Bogazici University, 34342 Istanbul, Turkey; nugay@boun.edu.tr

**Keywords:** self-healing, supramolecular polymer, hydrogen bonding, polydimethylsiloxane, polyurethane, vanadium pentoxide, graft copolymer, nanocomposite

## Abstract

The self-healing ability can be imparted to the polymers by different mechanisms. In this study, self-healing polydimethylsiloxane-graft-polyurethane (PDMS-*g*-PUR)/Vanadium pentoxide (V_2_O_5_) nanofiber supramolecular polymer composites based on a reversible hydrogen bonding mechanism are prepared. V_2_O_5_ nanofibers are synthesized via colloidal route and characterized by XRD, SEM, EDX, and TEM techniques. In order to prepare PDMS-*g*-PUR, linear aliphatic PUR having one –COOH functional group (PUR-COOH) is synthesized and grafted onto aminopropyl functionalized PDMS by EDC/HCl coupling reaction. PUR-COOH and PDMS-g-PUR are characterized by ^1^H NMR, FTIR. PDMS-*g*-PUR/V_2_O_5_ nanofiber composites are prepared and characterized by DSC/TGA, FTIR, and tensile tests. The self-healing ability of PDMS-graft-PUR and composites are determined by mechanical tests and optical microscope. Tensile strength data obtained from mechanical tests show that healing efficiencies of PDMS-*g*-PUR increase with healing time and reach 85.4 ± 1.2 % after waiting 120 min at 50 °C. The addition of V_2_O_5_ nanofibers enhances the mechanical properties and healing efficiency of the PDMS-*g*-PUR. An increase of healing efficiency and max tensile strength from 85.4 ± 1.2% to 95.3 ± 0.4% and 113.08 ± 5.24 kPa to 1443.40 ± 8.96 kPa is observed after the addition of 10 wt % V_2_O_5_ nanofiber into the polymer.

## 1. Introduction

In recent years, self-healing polydimethylsiloxane (PDMS) elastomers became one of the most appealing polymers as biomedical devices, flexible electronics, and actuators due to their good chemical stability, biocompatibility, and flexibility [1,2,3,4,5,6,7,8]. However, PDMS elastomers have restricted the ability to recover their mechanical and electrical properties via covalent bonding after damage [6,9]. In many studies, to overcome this problem researchers tried to enhance the self-healing properties of PDMS with supramolecular systems [7,8,10,11,12,13].

In supramolecular systems; the non-covalent interactions such as π-π interaction [11,14,15,16], ionic bonds [17], hydrogen bonding [8,18,19,20] and metal ligand interactions [21,22,23] are origins of self-healing behavior. Among various self-healing mechanisms in supramolecular polymers, healing through hydrogen bonding has attracted most attention by many research groups because hydrogen bonds can be easily separated and reconnected reversibly at room temperature, and the recovery properties can be easily adjusted via altering the number of hydrogen bonds [24,25,26,27]. 

Zhang and co-workers synthesized novel rubberlike, self-healable supramolecular elastomers by the reaction of multi acid functional polydimethylsiloxanes (SESi), diethylene triamine, and urea with a two-stage procedure. It was found that when the specimens having a polymerization degree of two were brought together just after they have been cut, tri–COOH terminated SESi showed more than 400% elongation via hydrogen rearrangement. However, healing efficiency of SESi decreases as the waiting time before reattachment increases. The authors claimed that this depends on the free and non-associated hydrogen bond units and as the time of attachment increases, the time for reassociation of hydrogen bonds increases [18,28]. In their other study, Zhang et al. investigated the crosslinking mechanism and hydrogen-bonding interactions of SESis in detail to control the synthesis and tune the polymer properties. The investigation shows that linking mechanism of SESis does not only consist of hydrogen bonds but also contains covalent crosslinking, and both hydrogen bonds and covalent crosslinking need to be controlled to obtain a polymer with desirable mechanical and healing properties [29]. Self-healing thermoplastic silicone elastomer having hydrogen bonding groups was synthesized by reacting the monomethacryloxypropyl terminated PDMS (PDMSMA) and 6-methyl-2-ureido-4[1H]-pyrimidone-bearing methacrylate (UPyMA) and magnetic Fe_3_O_4_ particles at different amounts, were incorporated into the elastomer by Skov and coworkers. Due to the presence of magnetic Fe_3_O_4_ particles, inductive heat was generated with an application of alternative magnetic field, which improved the self-healing properties of the copolymer significantly without any change in the mechanical properties [30]. Liu et al. modified hydrogen bonded triggered self-healable PDMS based elastomers with ureidopyrimidinone (UPy) to provide water to enhance healing and examined the effect of water on the properties of self-healing through hydrogen bonding motifs. UPy-NCO-PDMS were synthesized with the reaction of PDMS and UPy using hexamethylene diisocyanate (HMDI). It was indicated that the water medium acted as a plasticizer and endowed the reassembly of hydrogen bonds between UPy groups and accelerated the self-healing process of the supramolecular polymer [2]. 

Polyurethane (PUR) polymers that consist of hard and soft segments in their structure are another important polymer used for self-healing materials [31,32]. The formation of healable systems was enabled through soft segments providing mobility to PUR’s chains via an interaction of hydrogen bonds. However, an increase in the percentage of the hard segment hinders the self-healing properties of PUR [32,33]. Healing properties of PUR based polymers were tuned by altering the soft and hard segment throughout the polymer for a wide range of applications [34]. Jian et al. studied the self-healing properties of PUR by emphasizing the effect of disulfide and hydrogen bonds. They mentioned that hydrogen bonds enhance both self-healing properties and mechanical properties in early stages. In the case of disulfide bonds, the healing process needs a longer time for recovery, but disulfide bonds contribute to improving mechanical properties [32]. 

Some researchers found out that combining of PMDS and PUR played an advantageous role in improving the self-healing properties [1,3,21]. Whereas, to the best of our knowledge, there is limited research on the change of healing properties due to the addition of a different reinforcement in PDMS or modified PDMS supramolecular polymers [35,36]. It is also known that the addition of fillers to polymeric systems mostly improves their mechanical properties [37,38]. Bao and co-workers incorporated micro-nickel particles to a self-healable polymer containing amide/urea groups and proved that addition of nickel particles not only improves the mechanical properties but also enhances the self-healing properties by increasing the number of hydrogen bonds [9]. Different forms of vanadium pentoxide (V_2_O_5_) that are used in many applications such as sensors [39], actuators [40], and batteries [41,42] are also preferred as additives in polymers, especially to improve the electrical properties [43,44]. However, no experimental work has been reported regarding the effect of V_2_O_5_ on the self-healing properties of polymers. In one of the studies, it was stated that V_2_O_5_ paper was produced via disassociation of hydrogen groups [45]. 

In this study, aliphatic polyurethane grafted polydimethylsiloxane (PDMS-g-PUR) was synthesized as a self-healable supramolecular polymer and different amounts of V_2_O_5_ fibers were incorporated to improve the mechanical and self-healing properties through a reversible hydrogen bonding mechanism. The effect PUR grafting to PDMS and the addition of different amounts of V_2_O_5_ nanofibers on the thermal, mechanical, and healing properties of supramolecular polymer and composites were investigated.

## 2. Experimental Section

### 2.1. Materials and Chemicals

Hexamethylene diisocyanate (HMDI), polyethylene glycol (PEG) (*M*_n_: 400 g/mol), dibutyltin dilaurate (DBTDL), dimethyl sulfoxide (DMSO), anhydrous tetrahydrofuran (THF), anhydrous ethanol (EtOH), anhydrous citric acid (CA), *N*-(3-Dimethylaminopropyl) *N*′-ethylcarbodiimide hydrochloride (EDC HCl), and ammonium metavanadate and Dowex 50WX8, were purchased from Sigma-Aldrich Chemical Corporation (Taufkirchen, Germany). Poly[dimethylsiloxane-*co*-(3-aminopropyl)methylsiloxane] (AP-PDMS) was obtained from Gelest (Morrisville, PA, USA). All chemicals except PEG were used as received. PEG was purified by azeotropic distillation in toluene and stored under vacuum at 50 °C before each polymer synthesis. 

### 2.2. Synthesis

#### 2.2.1. Vanadium Pentoxide Nanofiber Synthesis

Vanadium pentoxide (V_2_O_5_) nanofibers were synthesized by a colloidal solution method as described in the literature [45]. Briefly, the V_2_O_5_ colloidal solution was prepared by dissolving 1 g of ammonium metavanadate in 200 mL deionized water and adding 10 g of acidic ion-exchange resin (Dowex 50WX8, Taufkirchen, Germany) into the solution. The colloidal solution was stirred for 10 min at 80 °C and cooled slowly (≈0.5 °C/min) to room temperature. After the resin was filtered, the solution was dried at room temperature. Subsequently, the dried nanofiber cluster was calcined at 150 °C in an air atmosphere under current humidity conditions to form a stable V_2_O_5_ phase.

#### 2.2.2. Synthesis of Polyurethane Having One –COOH Functional End Group

**Scheme 1:**
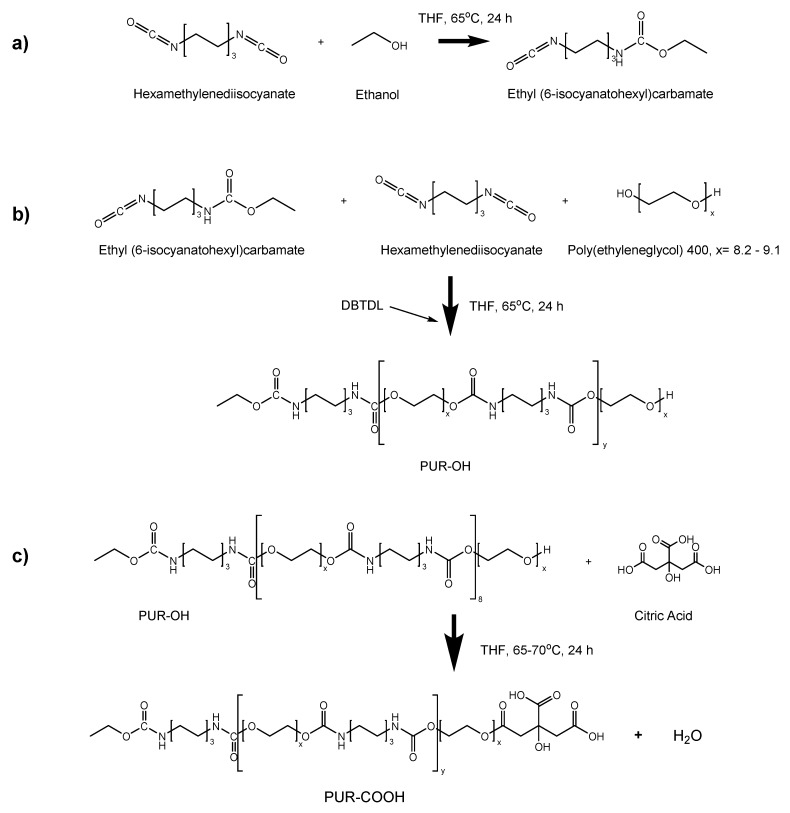
Synthesis of polyurethane (PUR)-COOH (**a**) capping of HMDI; (**b**) synthesis of PUR-OH; (**c**) esterification of PUR-OH with citric acid.

In order the synthesize polyurethane having one –COOH end group (PUR-COOH), linear aliphatic polyurethane having one –OH group (PUR-OH) was synthesized by step growth polymerization using DBTDL as a catalyst and then a –OH functional group was converted to a –COOH group using citric acid as illustrated in Scheme 1. First, one end group of some HMDI molecules were capped with ethanol to obtain ethyl 6-isocyanatohexyl carbamate via dissolving 0.445 mmol HMDI in 100 mL anhydrous THF under an argon atmosphere and then adding 4 µL of anhydrous ethanol into the solution dropwise (Scheme 1a). Dried PEG400 (0.5 mmol) was diluted with 100 mL anhydrous THF in another flask and transferred to a reaction flask under argon via cannulation. To initiate the polymerization, 0.03 wt % DBTDL was added into the reaction flask which contained PEG 400, HMDI, and ethyl 6-isocyanatohexyl carbamate. The polymerization was carried out under dry argon and stirred continuously for 48 h at 65 °C. (Scheme 1b). The PUR-OH was precipitated by the mixture into 800 mL cold hexane to precipitate the polymer. After filtration, the precipitate was washed five times with cold hexane to remove residual monomers and oligomers. The PUR-OH was dried in a vacuum oven at 40 °C for 24 h. The –OH of PUR-OH was converted to –COOH group via the esterification reaction with citric acid. The amount of hydroxyl end-groups in PUR-OH was calculated by acetic anhydride acetylation according to the ASTM E222 method, which is used to calculate the amount of citric acid needed to convert all the –OH groups to –COOH. It was found that the amount of citric acid was 200 mg. Based on the calculation, 1 g PUR-OH and 200 mg of citric acid was dissolved in THF under dry argon, separately. The solutions were mixed (Scheme 1c) and stirred vigorously under reflux at 65–70 °C. To determine the optimum reaction time, the unreacted citric acid concentration in solution was calculated by acid-base titration and no change in citric acid concentration was observed after 45 h. After 48 h, PUR-COOH was precipitated in 800 mL hexane and washed with cold methanol to eliminate residual citric acid. The product (PUR-COOH) was a translucent viscous yellow liquid and dried in a vacuum oven at 40 °C for 24 h and stored in a refrigerator until use. 

#### 2.2.3. Grafting of PUR-COOH onto AP-PDMS

**Scheme 2:**
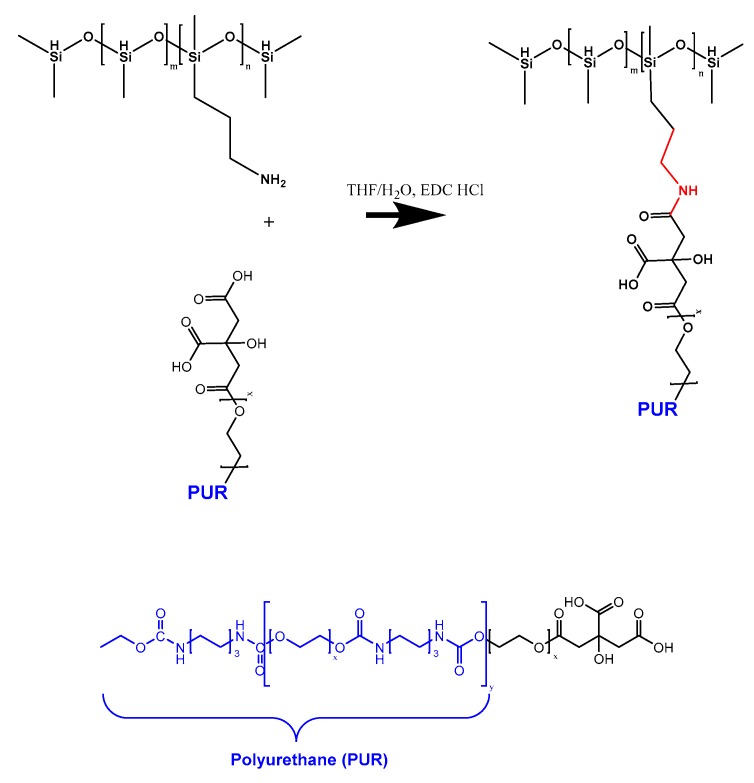
Synthesis of polydimethylsiloxane-*graft*-polyurethane (PDMS-*g*-PUR).

The grafting of PUR-COOH onto AP-PDMS was accomplished through carbodiimide coupling reaction using water soluble EDC. HCl as shown in Scheme 2. According to coupling procedure [46], 62 mmol EDC was dissolved in THF:H_2_O (4:1; *v*:*v*) and 66.7 mmol APPDMS was dissolved in THF at room temperature, separately. Then, the solutions were mixed and stirred in a three-necked round bottom flask under reflux at room temperature for 1 h. Then, 0.3 mmol PUR-COOH was added into the reaction mixture. The mixture was stirred continuously at 60 °C until the polymer precipitated at the bottom of the flask. The gel like polymer was separated from the solution and washed with THF. The synthesized graft copolymer (PDMS-*g*-PUR) was dried in a vacuum oven at 40 °C for 48 h. 

#### 2.2.4. Preparation of PDMS-*g*-PUR /V_2_O_5_ Composites

To prepare PDMS-*g*-PUR/V_2_O_5_ composites, 5 g PDMS-*g*-PUR was dissolved in 100 mL DMSO at 60 °C for 48 h. V_2_O_5_ nanofibers were dispersed in 100 mL of DMSO (Figure S1) using a SONICS Vibro-cell ultrasonic horn. The V_2_O_5_ nanofibers mixtures having different amounts of V_2_O_5_ as shown in Table 1 were mingled with PDMS-*g*-PUR solutions in an ultrasonic bath using a mechanical stirrer for 30 min at room temperature. Homogeneous composite mixtures were concentrated using ISOLAB rotary evaporator at 70 °C. The resultant viscous mixtures (approximately 20 wt % solid content) were poured into a Petri dish and then dried in a vacuum oven at 60 °C for 24 h. The dried samples were purified with acetone in a Soxhlet apparatus for 2 h, to remove the residual DMSO in the PDMS-*g*-PUR/V_2_O_5_ composites. All PDMS-*g*-PUR/V_2_O_5_ composites were dried at 40 °C for 12 h under vacuum. Afterwards, the PDMS-*g*-PUR/V_2_O_5_ supramolecular polymer composites were stored in a desiccator under argon until further characterization.

### 2.3. Characterization 

#### Characterization of V_2_O_5_ Nanofibers

The V_2_O_5_ nanofibers and residual phase such as VO and amorphous phases were investigated by XRD analysis. The evaluation of phases was compared for V_2_O_5_ nanofibers dried at room temperature and calcined at 150 °C. Powdered specimens were scanned from 2*q* = 10–90°, at a scanning rate of 2°/min, using a Rigaku DMax 2200 (Tokyo, Japan) diffractometer (with CuK_λ_-radiation, λ = 0.154 nm) at 40 kV and 40 mA. SEM and EDX analysis were performed to the calcined V_2_O_5_ nanofibers. A Phillips XL30 SFEG (North Billerica, MA, USA) scanning electron microscope was used for microstructural examination of samples with Secondary Electron Images, SEI, used predominantly, and distribution of elements identified with an energy dispersive X-ray spectrometer, EDX (North Billerica, MA, USA). Representative SEM images and spectrum were presented. Calcined V_2_O_5_ nanofibers were dispersed in methanol and a drop of the mixture was placed onto carbon coated copper grids to analyze the formation by TEM (Tecnai, G^2^ F20 S-TWIN, Columbus, OH, USA) analyses. The EDX detector of the TEM analyzer was used to verify the presence of elements.

### 2.4. Characterization of Polymer and Composites

Gel Permeation Chromatography (GPC) was used to determine the molecular weight of PUR-COOH. GPC experiments were carried out in THF at room temperature with 1 mL/min flow rate using an Agilent 1100 GPC (Santa Clara, CA, USA). The number average molecular weight and polydispersity index of PUR-COOH was 6011 g/mol and 2.39 respectively. Fourier-transform infrared spectroscopy (Digilab Excalibur FTS 3000, Randolph, MA, USA) and ^1^H NMR (a Varian NMRS-500 nuclear magnetic resonance instrument (500 MHz), West Sussex, UK) were used for chemical analyses of PUR-COOH and PDMS-*g*-PUR. FTIR analysis was performed between 4000–400 cm^−1^ at room temperature with an ATR set-up. ^1^H NMR spectra were recorded using deuterated DMSO or chloroform (CDCl_3_) as a solvent. Differential scanning calorimetric (DSC) analyses were carried out to obtain glass transition temperatures (*T*_g_) of pristine polymer and polymer composites. A DSC-TA instrument Q200 (TA Instruments, New Castle, DE, USA) was used under a nitrogen environment and the samples (each ~5 mg) were scanned from −80 °C to 100 °C with 10 °C/min ramp rate. Thermogravimetric analysis (TGA) were performed with a TGA-TA instrument 50 (TA Instruments, New Castle, DE, USA) under elevated temperature and nitrogen environment with a flow rate of 40 mL/min. An Instron (Noorwood, MA, USA) 5567 model universal electromechanical test machine was used to measure the mechanical properties of the pristine polymer and polymer composites as well as those that had been damaged and subsequently healed. The crosshead speed was 5 mm/min and a 100 N load cell was used. Six dog-bone shape specimens were tested based on ASTM 638 Type V standard for each composition. Mean values obtained from tested six specimens were reported.

Healing experiments were carried out at 50 °C. The samples were cut through the center with a sharp zirconia blade and then the pieces were brought together. This process was repeated three times and the change in the percentage of healing was examined after each time. In addition, the change of strength of healed samples was monitored as a function of time. After performing a tensile test on pristine and healed samples, the healing efficiency was calculated, which was defined as follows:

(1) $$Healing\;Efficiency\;\left(\%\right)=\frac{\sigma_{healed}}{\sigma_{pristine}}\times 100$$

where *σ_pristine_* and *σ_healed_* are the tensile strength of pristine and healed samples at the rupture. In addition, the healing behavior of the damaged regions was visualized via optical microscope (Olympus BX51, Center Valley, PA, USA) imaging at 50 °C (achieved with the use of a heating apparatus) as they underwent self-healing at 50 °C (with heating performed using a heating apparatus).

## 3. Results and Discussion

**Figure 1:**
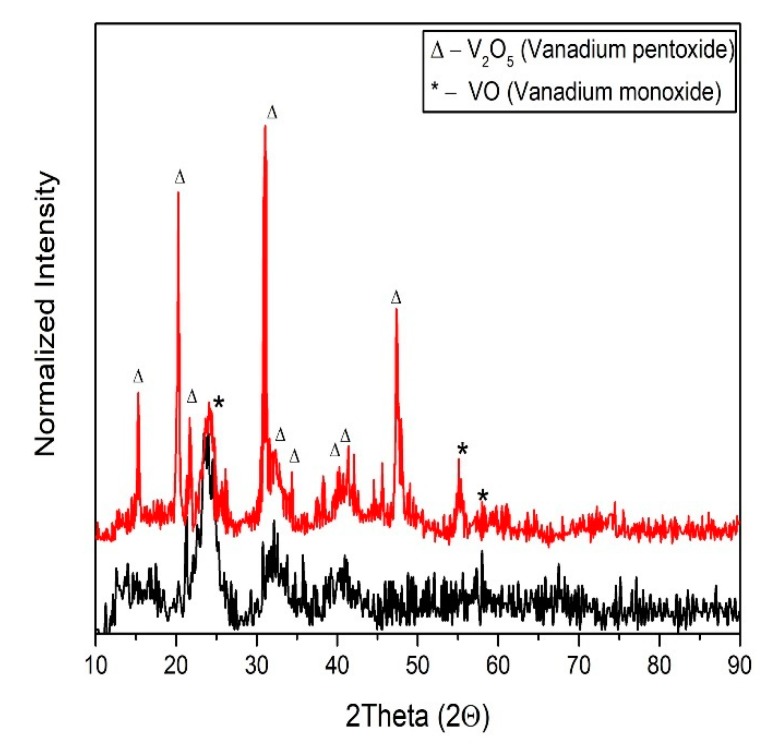
XRD patterns of V_2_O_5_ at room temperature (**black**) and at 150 °C (**red**).

The colloidal solution method described in the literature [45] was used to synthesize V_2_O_5_ nanofibers. The obtained V_2_O_5_ nanofibers were characterized by XRD, SEM, and TEM analyses. The presence of V_2_O_5_ phases at different temperatures was studied by XRD technique. Figure 1 shows the X-ray diffraction patterns of V_2_O_5_ nanofibers as a function of temperature. No considerable diffraction peak is observed in the pattern of V_2_O_5_ dried at room temperature (Figure 1-black line) pointing out that it is more likely an amorphous phase. After the calcination at 150 °C, the distinct peaks at 15.29°, 20.26°, 21.63°, 31.03°, and 47.41° were observed. These distinct peaks indicate the existence of a V_2_O_5_ crystal phase as a major phase. Besides the major V_2_O_5_ crystalline phase, peaks belonging to the VO phase can be also seen as a residual phase. (Figure 1-red line). 

**Figure 2:**
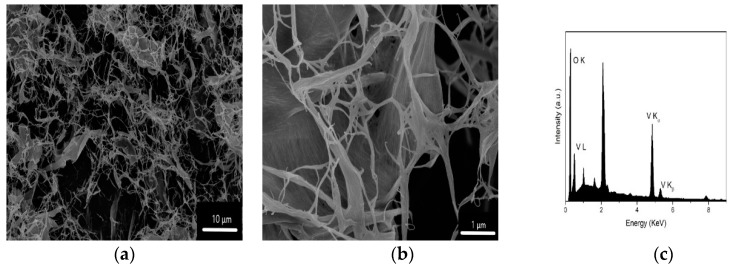
SEM images (**a**) and (**b**) and selected area EDX analysis (**c**) of V_2_O_5_ nanofiber clusters.

Figure 2a,b show the SEM images of V_2_O_5_ nanofibers calcined at 150 °C. Representative SEM images were given at different magnifications such as 5000× and 50,000× in Figure 2a,b, respectively. SEM figures indicate that the morphology of the synthesized V_2_O_5_ nanofibers is fibrous. Formation of stacking fibers is clearly seen from the magnified SEM image (Figure 2b). Figure 2c represents the selected area EDX analysis of the nanofibers, which is shown in Figure 2b. Only O and V elements are detected in EDX analysis indicating that nanofibers only consist of O and V elements without any other impurities (the data are given in Table S1) and this confirms the presence of V_2_O_5_ particles as consistent with XRD results. 

**Figure 3:**
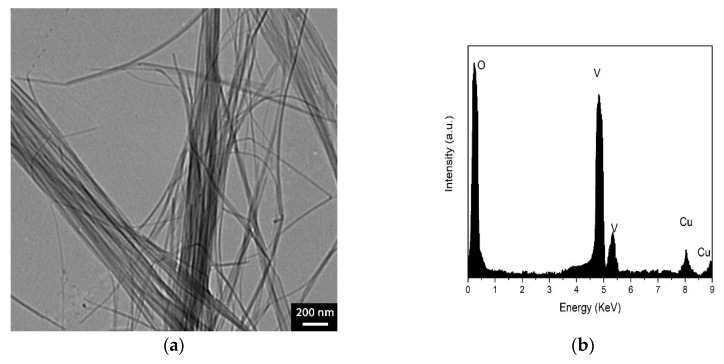
TEM image (**a**) and selected area EDX analysis (**b**) of V_2_O_5_ nanofiber cluster.

V_2_O_5_ nanofibers which were stacked after calcination at 150 °C were dispersed via sonication in the ultrasonic bath and then TEM analysis was conducted. The elongated dark places observed in the TEM image imply the long-range structure of V_2_O_5_ nanofibers (Figure 3a). EDX analysis (the data are given in Table S2) from TEM (Figure 3b) also confirms the evaluation of V_2_O_5_ nanofibers, which are only composed V and O elements. The TEM-EDX analysis results are in agreement with SEM-EDX analysis results. 

In order to prepare the PDMS-*g*-PUR copolymer, first linear aliphatic PUR having one –OH functional group (PUR-OH) was synthesized by a step polymerization reaction using DBTDL as a catalyst in THF. Figure S2 shows the ^1^H NMR spectrum of PUR-OH. The –OH functional group was then converted to –COOH group using citric acid to enable the synthesis of copolymer through EDC coupling reaction [46]. 

**Figure 4:**
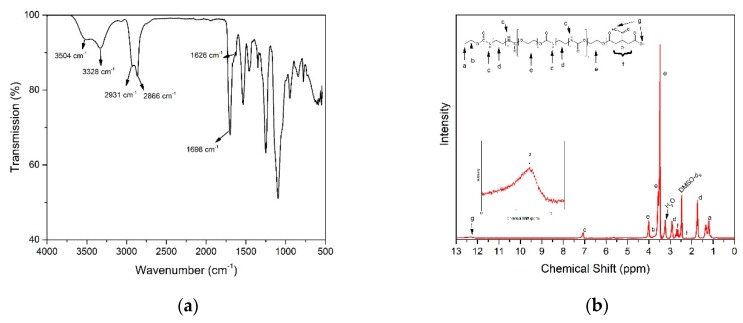
FTIR (**a**) and ^1^H NMR (**b**) spectra of PUR-COOH.

To investigate the chemical composition of PUR-COOH in detail, FTIR and ^1^H NMR analyses were performed. Figure 4a presents the FTIR spectrum of PUR-COOH. The vibrational bands at between 3680 cm^−1^ and 3120 cm^−1^ are due to O–H and N–H stretching and can be attributed to urethane linkage [33]. In addition, the presence of the C–H stretching at 2931 cm^−1^ and 2866 cm^−1^, C=O stretching at 1698 cm^−1^ and 1626 cm^−1^ vibrational bands also confirm the PUR formation [32,33,47]. 

Figure 4b shows the ^1^H NMR spectrum of PUR-COOH and confirms that the PUR chains have a –COOH end group. This analysis was carried out in deuterated DMSO to observe the –NH linkage more clearly. Both ^1^H NMR peaks obtained from the DMSO and residual H_2_O inside the DMSO can be seen from the spectra. The peak at 7.06 (c) ppm can be assigned to –NH which belongs to a urethane linkage. In addition, the peaks at 4.01 (e) ppm, 3.56 (e) ppm and 3.47 (e) ppm can be attributed to the polyethylene glycol monomer. The peak at 12.30 ppm shown with g corresponds to COOH groups. Thus, the results obtained from NMR spectra show consistency with FTIR results and indicate that the PUR-COOH synthesis was successful.

**Figure 5:**
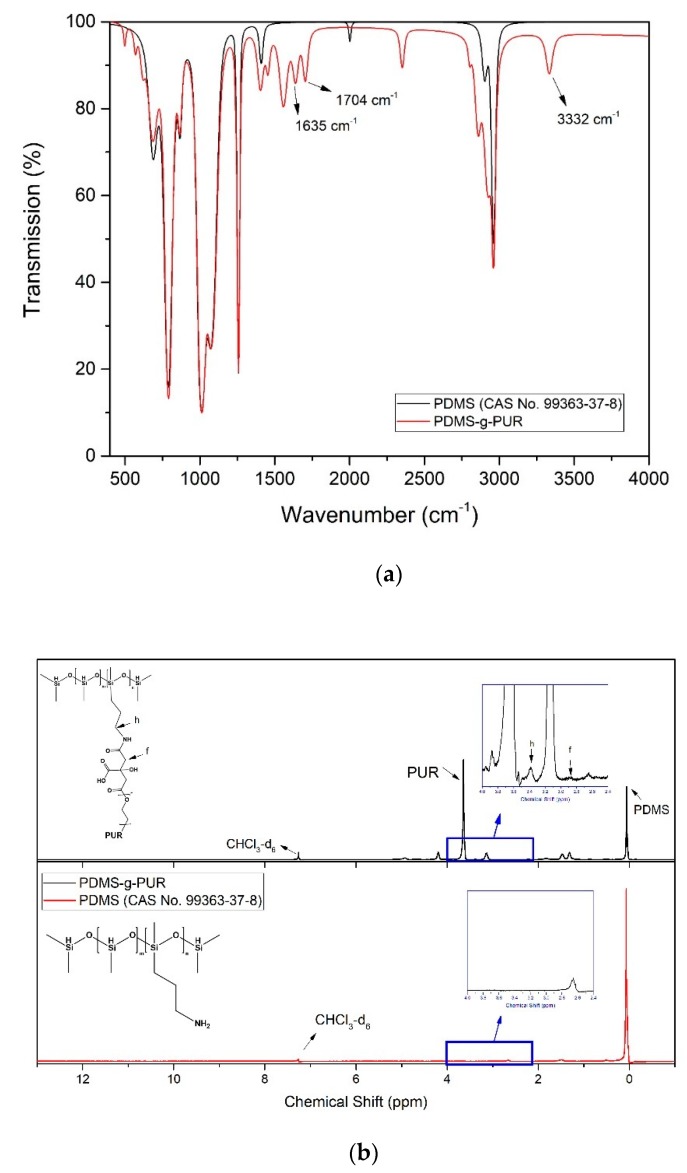
(**a**) Comparison of FTIR spectra of PDMS-*g*-PUR (**red**) and PDMS (**black**) and (**b**) ^1^H NMR spectra of PDMS-*g*-PUR and commercial PDMS (which is used to synthesize graft copolymer).

To synthesize the PDMS-*g*-PUR, the PUR-COOH was grafted onto aminopropyl functionalized PDMS by an EDC coupling reaction. To show the success of the grafting, the comparison between the FTIR and ^1^H NMR spectra of PDMS and PDMS-*g*-PUR are given in Figure 5a,b, respectively. When the two FTIR spectra are compared, the appearance of a new peak at between 3400–3250 cm^−1^ corresponding to an increase in N–H linkage in the polymeric system is seen after grafting. Three peaks between 1500–1700 cm^−1^ due to C=O and N–H stretching also appeared after graft polymer formation. The ^1^H NMR spectra of PDMS and PDMS-*g*-PUR are also compared, the peak at around 3.5 ppm, which is a characteristic peak corresponding to PEG groups in polyurethanes structure is observed in PDMS-*g*-PUR‘s spectrum. The peak near 0 ppm can be assigned to the H atoms in the –Si–O–CH_3_ group in the grafted polymer.

**Figure 6:**
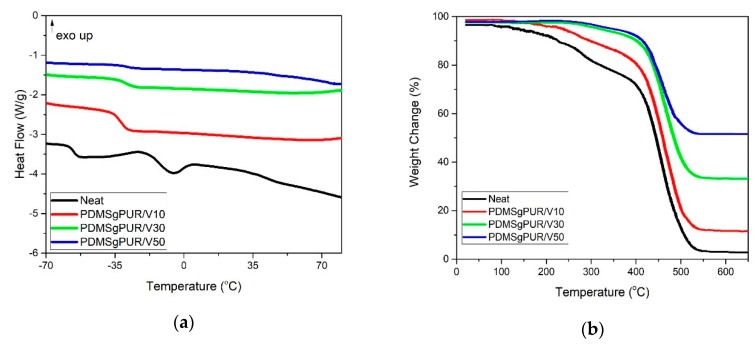
(**a**) Differential scanning calorimetric (DSC) curves of PDMS-*g*-PUR (**neat**) and PDMS-*g*-PUR/V_2_O_5_ composites and (**b**) thermogravimetric analysis of PDMS-*g*-PUR (**neat**) and PDMS-*g*-PUR/V_2_O_5_ composites.

PDMS-*g*-PUR polymer was reinforced with V_2_O_5_ nanofibers to enhance the mechanical properties, and the effect of V_2_O_5_ nanofibers on the healing efficiency was investigated. Changes in *T*_g_ via DSC curves, thermal stability via TG analysis, mechanical properties, and healing properties via tensile test and the optical microscope were determined. Figure 6a illustrates DSC heating curves for PDMS-*g*-PUR/V_2_O_5_ composites. Results of PDMS-*g*-PUR/V_2_O_5_ composites were compared with the result of PDMS-*g*-PUR to determine the effect of V_2_O_5_ on the polymers’ glass transition temperature (*T*_g_). The *T*_g_ values of neat polymer and polymer composites are given in Table S3. As shown in Figure 6a, two different *T*_g_s were detected at −53.1 °C and −12.5 °C for the PDMS-*g*-PUR polymer. It is observed that with a 10 wt % V_2_O_5_ nanofiber addition to the polymeric system, the *T*_g_ at −12.5 °C disappeared and *T*_g_ at −53.2 °C increased to −31.4 °C with a 41% increment. When the percentage of reinforcement further increased, the change in the *T*_g_ was slowed down and this can be explained by the maximum loading capacity of PDMS-*g*-PUR of V_2_O_5_ [43]. 

Thermal properties obtained from TGA curves for PDMS-*g*-PUR and PDMS-*g*-PUR/V_2_O_5_ composites are given in Figure 6b. As can be seen from the TG curve, degradation of neat polymer started at around 100 °C. At 400 °C, 28.1 wt % weight lost is observed. With a 10 wt % V_2_O_5_ addition, polymer composite decomposition temperature increases to 150 °C and 20 wt % of the polymer composite degrades at 400 °C. The thermal stability of the polymer increases after 10 wt % V_2_O_5_ addition due to the restriction of chain mobility [43]. When the amount of nanofibers in the polymer composite is 30 wt %, a further increase in decomposition temperature up 270 °C is observed and total weight lost at 400 °C is found to be 10.9 wt %, indicating as the amount of V_2_O_5_ nanofibers increases, improvement in the thermal stability of PDMS-*g*-PUR/V_2_O_5_ polymer composites is observed. In the case of 50 wt % V_2_O_5_ nanofiber reinforced polymer composites, no considerable change in decomposition temperature is observed as compared to the polymer composite containing 30 wt % V_2_O_5_, which can be ascribed to the maximum loading capacity of polymer composite and 8.3 wt % weight lost is seen at 400 °C, which is consistent with DSC results. Above 400 °C decomposition was increased enormously for both neat polymer and polymer composites. 

**Figure 7:**
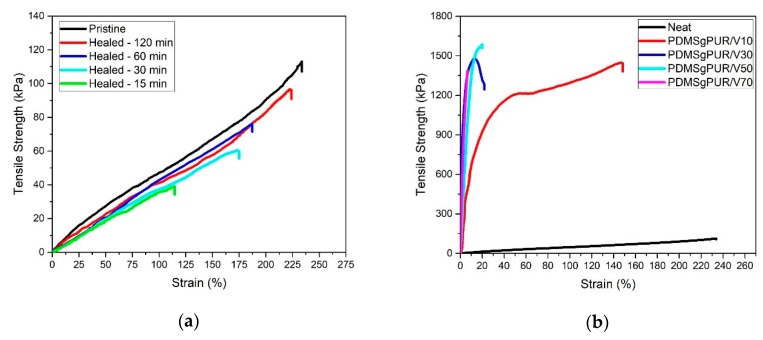
(**a**) Tensile test results of PDMS-*g*-PUR and healed PDMS-*g*-PUR as a function of healing time (min) and (**b**) tensile test results of PDMS-*g*-PUR and PDMS-*g*-PUR/V_2_O_5_ composites.

The mechanical behavior and healing efficiencies of PDMS-*g*-PUR and PDMS-*g*-PUR/V_2_O_5_ polymer composites are characterized via tensile test. First, the rectangular specimens of the pristine PDMS-*g*-PUR polymer were cut into two pieces. Subsequently, the cut pieces were brought and held together for different times (15, 30, 60, and 120 min) at 50 °C to determine the healing efficiency through tensile strength obtained from the tensile test. Healing efficiency was calculated from the tensile strength ratio of healed specimens to the pristine polymer or polymer composites. Figure 7a presents the stress-strain curve of pristine PDMS-*g*-PUR and healed PDMS-*g*-PUR for different healing times. The maximum tensile strength of the pristine polymer is found to be 113.08 ± 5.24 kPa, and the healing efficiency is 31.8 ± 2.7% for the specimens held for 15 min with a 34.51 ± 2.36 kPa tensile strength. When the cut pieces are held together for a longer time, an increase in their maximum stress values and the healing efficiencies are observed. The maximum tensile strength and healing efficiency are found to be 96.52 ± 4.54 kPa and 85.4 ± 1.2%, respectively, for 120 min holding time.

After the mechanical properties and healing efficiency of the PDMS-*g*-PUR polymer were determined, V_2_O_5_ nanofibers were incorporated into the PDMS-*g*-PUR to improve both mechanical properties and healing efficiencies via formation of the PDMS-*g*-PUR /V_2_O_5_ polymer composites. All the specimens for polymer composites were brought together at 50 °C for 120 min after the cut to observe the healing process. Figure 7b compares the stress-strain curve of pristine PDMS-*g*-PUR with polymer composites loaded with different amounts of V_2_O_5_ nanofibers up to 70 wt %. The maximum stress increases to 1443.40 ± 8.96 kPa with an addition of 10 wt % V_2_O_5_ nanofibers whereas strain value of PDMS-*g*-PUR/V_2_O_5_ composites at maximum stress decreases from 246.82 ± 16.3% to 148.37 ± 4.78% due to the restriction of chain mobility after the addition of 10 wt % V_2_O_5_ nanofibers [43]. When the amount of V_2_O_5_ addition increases to the 30 wt % and 50 wt %, the tensile strength of composites slightly increases up to 1479.21 ± 17.22 kPa and 1584.38 ± 18.12 kPa, respectively. Nevertheless, with an addition of 70 wt % V_2_O_5_ nanofibers, the strength of composite decreases to 1384.00 ± 21.4 kPa and this can be due to the agglomeration of an excessive amount of V_2_O_5_ nanofibers inside the polymer matrix. Tensile strain value decreases to 13.05 ± 1.2% after the addition of 30 wt % V_2_O_5_ nanofibers, and with a further increase in the amount of nanofibers, no considerable change in tensile values is observed due to maximum loading capacity as detected in the DSC and TGA results. 

**Figure 8:**
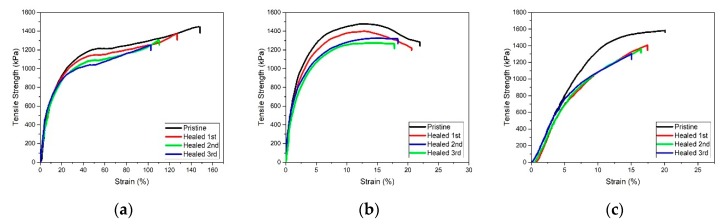
Tensile test results of PDMS-*g*-PUR composites and healed samples (**a**) PDMS-*g*-PUR/V10 (**b**) PDMS-*g*-PUR/V30 and (**c**) PDMS-*g*-PUR/V50. (The samples were healed at 50 °C for 2 h)

In order to see the effect of V_2_O_5_ nanofiber addition into PDMS-*g*-PUR and V_2_O_5_ nanofiber amount on healing efficiencies, pristine polymer reinforced with 10, 30, and 50 wt % V_2_O_5_ nanofibers cut into two pieces were brought together and held for 120 min at 50 °C, and this process was repeated one, two, and three times, respectively. After each cycle of reattachment, specimens were subjected to the tensile test and their first, second, and third healing efficiencies were determined. Figure 8a–c show the stress-strain curves of all pristine and healed specimens. In Figure 8a, stress-strain curves of PDMS-*g*-PUR /10V are shown before and after healing cycles. Healing efficiency of PDMS-*g*-PUR/10V is found to be 95.3 ± 0.4% after the first self-healing cycle. In the supramolecular systems, the healing is mostly generated with the reformation of unbounded hydrogen bonds moieties [8,9,18,19,20,21]. Bao et al. synthesized synthesis supramolecular hydrogen bonding network polymer composites that were reinforced with micro-nickel particles. In their study, they prepared a composite that had similar mechanical and thermal behavior whereas with less hydrogen bonding sites compared to their original composites to evaluate the effect of hydrogen bonding on healing properties of supramolecular systems. They found that the healing efficiency of the composite having less hydrogen bonding sites decreased significantly to 7% compared to their original composites, which had 90% healing efficiency [9]. 

Burghard et al. showed that the hydrogen bonding sites placed at the surround of V_2_O_5_ fibers [45]. With a 10 wt % V_2_O_5_ nanofiber addition, an increase in the healing efficiency of PDMS-*g*-PUR /V10 polymer composite compared to the PDMS-*g*-PUR (Figure 8a) was observed. It is also shown in the literature that the hydrogen bonding effects the self-healing properties [9], therefore an increase in the healing efficiency can be attributed to the increase in the number of available hydrogen bonding sites with V_2_O_5_ nanofiber addition. When the amount of V_2_O_5_ nanofiber is increased to 30 wt % and 50 wt %, the healing efficiencies after the first cycle are determined as 94.7 ± 0.5% and 88.9±0.9%, respectively as seen in Figure 8b,c. It can be clearly seen that the healing efficiencies of composites decrease with an increase in V_2_O_5_ nanofiber content. The reason for this decrease may be due to the reduction of the contact surface required for the hydrogen bonding, as the amount of V_2_O_5_ nanofiber additive increases in the polymer composite [15]. Figure 8a–c also illustrate that healing efficiencies decrease when the number of healing cycle increases. The healing efficiencies are 86.7 ± 0.5%, 86.1 ± 0.4%, and 82.3 ± 1.8% for 10, 30, and 50 wt % V_2_O_5_ nanofibers reinforced PDMS-*g*-PUR/V_2_O_5_ composites after the specimens are cut and brought together three times, respectively (Table 2).

**Figure 9:**
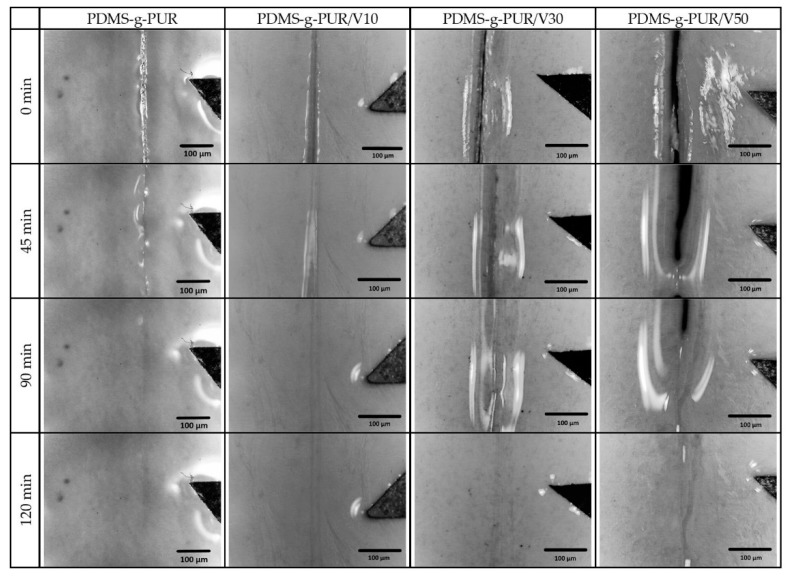
The optical microscope images of self-healing behavior of knife-cut PDMS-*g*-PUR and PDMS-*g*-PUR /V_2_O_5_ composites as a function of healing time. The healing was done at 50 °C.

The self–healing behavior of neat polymer and composites was also observed via optical microscope. A film of neat polymer and polymer composites containing different amounts of V_2_O_5_ nanofiber were prepared and a small cut was made on a surface with a knife, subsequently allowing it to undergo healing at 50 °C for different amounts of time, and then images were taken with an optical microscope, as can be seen in Figure 9. Figure 9 presents that healing after 90 min and 120 min in the damaged area is observed for neat PDMS-*g*-PUR and PDMS-*g*-PUR/V10 composite, respectively. Damaged areas belong to PDMS-*g*-PUR/V30 and PDMS-*g*-PUR/V50 polymer composites are able to repair the notch after 120 min but cuts do not fully disappear as seen from Figure 9. From the optical microscope images, it can be said that all the specimens are healed after 120 min, however, the composites consisting of more V_2_O_5_ nanofiber content heal slower due to the decrease in the chain motion, which is consistent with tensile results. Although the composites heal slower than neat polymer, the healing efficiencies of fully recovered composites are found to be greater due to the contribution of V_2_O_5_ nanofibers to hydrogen bonding capability, as indicated in the tensile strength results.

## 4. Conclusions

A self-healable supramolecular polymer has been successfully prepared by grafting an aliphatic polyurethane to polydimethylsiloxane (PDMS-*g*-PUR). The PDMS-*g*-PUR has the maximum tensile strength of 113.08 ± 5.24 kPa and heals itself after waiting 2 h at 50 °C with a healing efficiency of 85.4 ± 1.2%. To improve the mechanical properties and healing efficiency of the PDMS-*g*-PUR, polymer composites incorporating V_2_O_5_ nanofibers which have the ability to make hydrogen bonds are prepared. The mechanical properties and healing efficiencies of PDMS-g-PUR are enhanced with 10 wt % V_2_O_5_ nanofiber addition. A decrease in total healing efficiencies is observed when the V_2_O_5_ nanofibers content is increased to 30 wt % and 50 wt % but they were still found to be greater than the healing efficiency of the neat PDMS-g-PUR. Addition of V_2_O_5_ nanofibers increases the hydrogen bonding capability of the polymer and reduces the contact surface required for the hydrogen bonding, leading to increased healing efficiencies compared to neat polymer. 

## Figures and Tables

**Table 1:** Composition of PDMS-*g*-PUR/V_2_O_5_ composites.

Composition Name	V_2_O_5_ (%)	PDMS-*g*-PUR (%)
PDMS-*g*-PUR	-	100
PDMS-*g*-PUR/V10	10	90
PDMS-*g*-PUR/V30	30	70
PDMS-*g*-PUR/V50	50	50
PDMS-*g*-PUR/V70	70	30

**Table 2:** Healing efficiencies of PDMS-*g*-PUR and PDMS-*g*-PUR/V_2_O_5_ composites.

	1st Healing (%)	2nd Healing (%)	3rd Healing (%)
PDMS-*g*-PUR	85.4 ± 1.2	-	-
PDMS-*g*-PUR/V10	95.3 ± 0.4	90.1 ± 0.9	86.7 ± 0.5
PDMS-*g*-PUR/V30	94.7 ± 0.5	89.6 ± 0.7	86.1 ± 0.4
PDMS-*g*-PUR/V50	88.9 ± 0.9	86.2 ± 1.5	82.3 ± 1.8

## Supplementary Materials

The following are available online at http://www.mdpi.com/2073-4360/11/1/41/s1, Figure S1: Vanadium pentoxide (V_2_O_5_) nanofiber (calcined at 150 °C) dispersed in DMSO, Figure S2: ^1^H NMR spectrum of PUR-OH polymer, Table S1: Chemical composition of V_2_O_5_ nanofiber obtained from SEM-EDX analysis, Table S2: Chemical composition of V_2_O_5_ nanofiber obtained from TEM-EDX analysis, Table S3: T_g_ values of PDMS-*g*-PUR and PDMS-*g*-PUR /V_2_O_5_ composites.
