# Supplementary material for: Self-Healable Supramolecular Vanadium Pentoxide Reinforced Polydimethylsiloxane-Graft-Polyurethane Composites
Source: Polymers (Basel). 2018 Dec 29;11(1):41. doi: 10.3390/polym11010041 (PMC6401741; doi:10.3390/polym11010041)
Supplement: Supplementary file 1 [file polymers-11-00041-s001.pdf]

Supplementary Materials: Self-Healable Supramolecular Vanadium Pentoxide Reinforced Polydimethylsiloxane-Graft-Polyurethane Composites

Ali Sabri Berkem, Ahmet Capoglu, Turgut Nugay, Erol Sancaktar and Ilke Anac

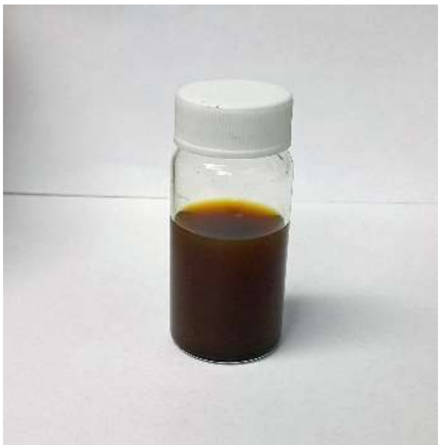

Figure S1. Vanadium pentoxide (V_2O_5) nanofiber (calcined at 150 °C) dispersed in DMSO.

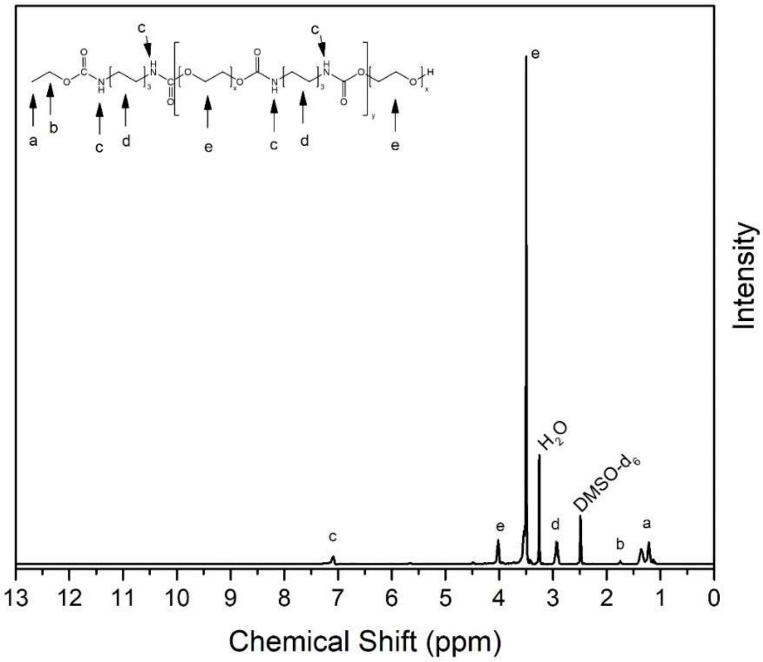

Figure S2. ^{1}H NMR spectrum of PUR-OH polymer.

Table S1. Chemical composition of V_2O_5 nanofiber obtained from SEM-EDX analysis.

Element	Weight %	Atomic %
O-K	26.14	52.98
V-K	73.86	47.02

Table S2. Chemical composition of V_2O_5 nanofiber obtained from TEM-EDX analysis.

Element	Weight %	Atomic %
O-K	36.14	64.65
V-K	63.52	35.35

Table S3. T_g values of PDMS-*g*-PUR and PDMS-*g*-PUR/V_2O_5 composites.

	T_{g1} (˚C)	T_{g2} (˚C)
PDMS-*g*-PUR	-53.1	-12.5
PDMS-*g*-PUR/V10	-31.4	-
PDMS-*g*-PUR/V30	29.4	-
PDMS-*g*-PUR/V50	28.2	-
